# Type VI secretion system-associated FHA domain protein TagH regulates the hemolytic activity and virulence of Vibrio cholerae

**DOI:** 10.1080/19490976.2022.2055440

**Published:** 2022-04-06

**Authors:** Guangli Wang, Chan Fan, Hui Wang, Chengyi Jia, Xiaoting Li, Jianru Yang, Tao Zhang, Song Gao, Xun Min, Jian Huang

**Affiliations:** a Department of Laboratory Medicine, Affiliated Hospital of Zunyi Medical University, Zunyi, Guizhou, China; b School of Laboratory Medicine, Zunyi Medical University, Zunyi, Guizhou, China

**Keywords:** *Vibrio cholerae*, forkhead-associated domain, TagH, type VI secretion system, hemolysin

## Abstract

The type VI secretion system (T6SS) and hemolysin HlyA are important virulence factors in *Vibrio cholerae*. The forkhead-associated (FHA) domain is a conserved phosphopeptide binding domain that exists in many regulatory modules. The FHA domain protein-encoding gene is conserved in the T6SS gene cluster and regulates the assembly and secretion of the T6SS. This study shows for the first time that the FHA domain protein TagH plays a role in controlling the hemolytic activity of *V. cholerae*, in addition to regulating the T6SS. TagH negatively regulates HlyA expression at the transcriptional and post-translational levels. The phosphopeptide binding sites of the FHA domain of TagH play a key role in the regulation of hemolytic activity. The deletion of *tagH* enhances the intestinal pathogenicity and extraintestinal invasion ability of *V. cholerae*, which mainly depend on the expression of HlyA. This study provides evidence that helps unravel the novel regulatory role of TagH in HlyA and provides critical insights which will aid in the development of strategies to manage HlyA.

## Introduction

*Vibrio cholerae* mainly exists in aquatic environments and can be transmitted to human hosts through the ingestion of contaminated food or water.^1^^,2^ Based on its surface O antigens, *V. cholerae* can be classified into more than 200 serotypes.^3^ Cholera epidemics or pandemics are only caused by the O1 and O139 serogroups, and the non-O1/non-O139 serogroups are mainly associated with gastroenteritis or parenteral infections, such as bacteremia,^4^ skin and soft tissue infections,^5^ and meningitis.^6,7^ Notably, non-O1/non-O139 *V. cholerae* bacteremia is a serious disease with high mortality, and is considered a global health threat, especially in patients with a history of alcohol abuse and/or cirrhosis. However, its pathogenic mechanisms are currently unclear. In the previous two decades, invasive infections caused by non-O1/non-O139 *V. cholerae* have increased, and are significantly higher than the number of reported O1 and O139 *V. cholerae* cases.^8^ The O1, O139, and non-O1/non-O139 *V. cholerae* strains have varying numbers of virulence factors, and this is thought to be the reason for the differences in their clinical results.^9,10^ The main pathogenic factors of O1 and O139 strains are cholera toxin (CT) and toxin-coregulated pili (TCP).^11,12^ In contrast, non-O1/non-O139 strains do not contain CT and TCP, and their virulence mechanisms are not yet well defined.^13,14^ However, additional virulence factors such as the type III secretion system (T3SS), heat-stabilized enterotoxin, hemolysin (HlyA), mannose-sensitive hemagglutinin, and the type VI secretion system (T6SS) have been identified in non-O1/non-O139 strains.^9,15^

*V. cholerae* HlyA is an important virulence factor that belongs to the pore-forming toxin family, produced in the El Tor biotype and most non-O1/non-O139 isolates.^16,17^ HlyA has vacuolar and cytocidal activity in many cell lines,^18,19^ and is associated with rapid mortality in mice.^20,21^ In addition, in non-O1/non-O139 *V. cholerae*, HlyA has been shown to be a contact-independent competitive factor in the ecological niches.^22^ The T6SS is another key contact-dependent weapon that can directly kill competitors through the translocation of effectors. *V. cholerae* was one of the first bacteria in which a T6SS was identified, and it has now been found in all sequenced isolates of *V. cholerae*.^2,23^ It is a multi-component toxin delivery device that is structurally and functionally analogous to the T4 bacteriophage tail spike.^24,25^ The T6SS not only plays an important role in inter- and intra-species competition in the aquatic environment and host life cycle but also contributes to virulence and intestinal colonization.^22,25–29^

The forkhead-associated (FHA) domain is a phosphopeptide binding module,^30,31^ which is found in many regulatory proteins in both eukaryotes and bacteria. However, the functions of the FHA domain proteins in bacterial physiology remain unclear. The FHA domain has previously been found to play a variety of roles in bacteria such as cell shape regulation, pathogenicity, host-bacterial interaction, and type III secretion.^31,32^ Interestingly, many of the T6SS gene clusters encode orthologs of the FHA-associated domain-containing proteins, serine-threonine kinase (PpkA) and serine-threonine phosphatase (PppA), which are involved in the regulation of threonine phosphorylation events. For example, in *Pseudomonas aeruginosa* and *Serratia marcescens*, PpkA and PppA regulate the threonine phosphorylation and dephosphorylation of FHA domain proteins, respectively, and this affects the assembly and secretion of the T6SS.^26,30,33,34^ However, genomic sequence analysis found that some bacterial T6SS gene clusters were contained only in the FHA domain protein coding genes without PpkA and PppA coding genes, such as those of *V. cholerae, E. coli* O157, and *Y. pestis*.^35,36^ The biological function of the FHA domain protein in these bacteria is not fully understood. Only one gene in the *V. cholerae* genome encodes the FHA domain protein, and it is present in the T6SS gene cluster; its encoded protein is annotated as the T6SS-associated FHA domain protein TagH, and it contains a 93 aa FHA domain (aa 8–110). Since the *ppkA* and *pppA* genes have not been found in the genome of *V. cholerae*, the function of TagH is currently unclear.^34,36^ It was also previously shown that T6SS hallmark protein Hcp cannot be secreted in the *VCA0112* (*tagH*) mutant, suggesting that TagH is required for the T6SS to function in *V. cholerae*.^37^ In this study, we found that TagH plays a previously unrecognized role in controlling *V. cholerae* hemolytic activity and virulence, in addition to regulating the T6SS.

## Results

### TagH represses hemolytic activity and cytotoxicity in V. cholerae

**Figure 1.**
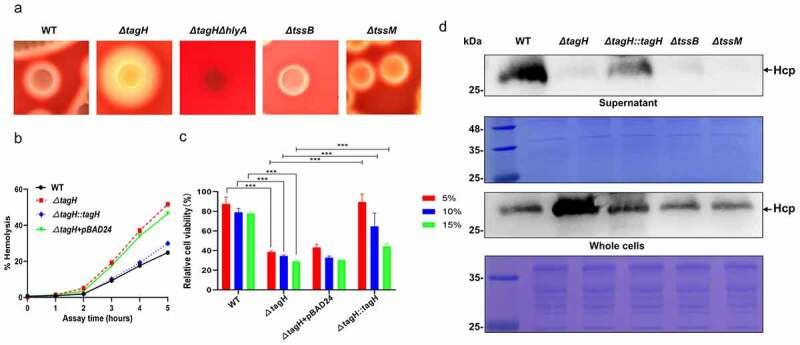
**TagH negatively regulates the hemolytic and cytotoxic activities of *V. cholerae*. a** The hemolytic zone of the wild-type (WT), *ΔtagH, ΔtagHΔhlyA, ΔtssB* and *ΔtssM* strains cultured on 5% sheep blood agar at 37°C for 24 h. **b** Hemolysis activity of the culture supernatants from the WT, *ΔtagH*, and complementary mutant *ΔtagH::tagH*. The culture supernatants and erythrocyte suspensions were mixed at a ratio of 9:1 and incubated at 37°C for 5 h. The supernatant was collected every hour to measure its absorbance at 540 nm. The relative hemolysis rates are shown as the means and standard errors of three biological replicates (n = 3). **c** Cytotoxicity of culture supernatants from various strains. SW480 cells were treated with WT, *ΔtagH* and *ΔtagH::tagH* culture supernatants with 5%, 10%, and 15% dilutions at 37°C for 8 h. The cell viability of the SW480 cells via the CCK-8 assay. The values are shown as means and standard errors of six biological replicates (n = 6). ***, *P* < .001 between two strains. **d** Western blotting analysis for the precipitated culture supernatants and whole cell lysates from the WT, *ΔtagH, ΔtagH::tagH, ΔtssB* and *ΔtssM* strains with anti-Hcp serum. A Coomassie blue stained gel was used to estimate the loading amount for each sample.

Previously, an FHA domain-containing protein was shown to contribute to Hcp secretion in the *V. cholerae* O37 serogroup strain V52^37^. In this study, a *tagH* mutant (*ΔtagH*) was constructed in the non-O1/non-O139 *V. cholerae* HN375 strain. Interestingly, we found that the hemolysis zone for the *ΔtagH* mutant on sheep blood agar was significantly larger than that for the wild-type strain (Figure 1a). Since *V. cholerae* lyses red blood cells by secreting hemolysin, we collected the culture supernatant of each strain to further investigate hemolysis. The results showed that the hemolytic activity of the *ΔtagH* strain was significantly higher than that of the wild-type (Figure 1b). The hemolytic activity of the complemented mutant *ΔtagH::tagH* was restored to the wild-type level (Figure 1b). The pBAD24 empty plasmid did not restore the hemolytic activity of the *ΔtagH* strain. These data suggest that TagH may be a negative regulator of *V. cholerae* hemolysin. *V. cholerae* hemolysin is also an important cytotoxin, as it has previously been shown to have cytocidal activity against a variety of cell lines.^38^ We further tested the cytotoxicity of the culture supernatant for each strain at different dilution ratios with the SW480 cells (human colon adenocarcinoma cells). The results showed that when compared with the lysogeny broth (LB) medium, the supernatant of the wild-type strain reduced cell viability, while that of the *ΔtagH* mutant significantly decreased cell viability when compared with the wild-type (Figure 1c). Compared with the *ΔtagH* group, the cell viability of the complemented mutant *ΔtagH::tagH* culture supernatant was restored to the wild-type level, while the pBAD24 plasmid had no replenishment effect. Taken together, these data support the hypothesis that TagH negatively regulates the hemolytic and cytotoxic activities of *V. cholerae*. To further examine the effects of TagH on the activity of the T6SS, we analyzed the expression of Hcp in the culture supernatant and whole cell lysates. We found that Hcp secretion was abolished in the *ΔtagH* mutant, while in the complemented mutant *ΔtagH::tagH* it was restored to a level similar to that of the wild-type strain (Figure 1d). In addition, the expression level of intracellular Hcp was significantly increased in the *ΔtagH* mutant, while in T6SS null mutants *ΔtssB* and *ΔtssM*, it was similar to that of the wild-type strain (Figure 1d). These results suggest that *tagH* knockout affects the secretion and intracellular expression of Hcp in the non-O1/non-O139 *V. cholerae* HN375 strain.

### Regulation of the hemolytic activity and cytotoxicity in V. cholerae by TagH depends on the expression of HlyA

**Figure 2.**
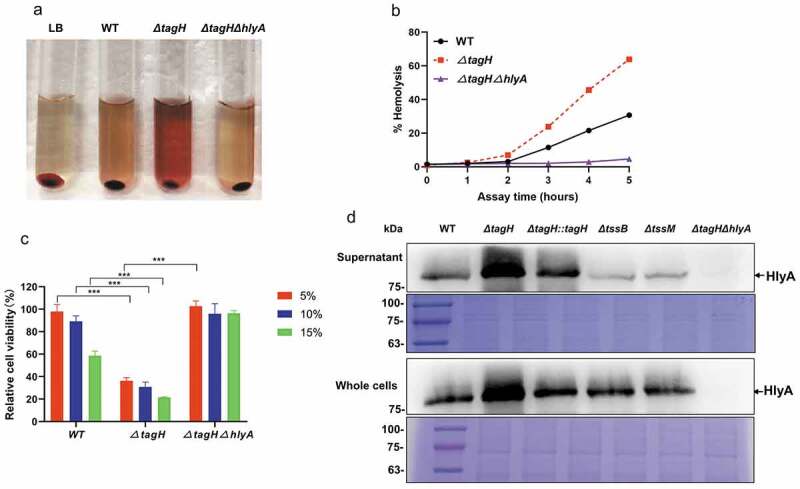
**TagH regulation of the hemolytic activity and cytotoxicity of *V. cholerae* depends on HlyA expression. a-b** Hemolytic activity analysis of the culture supernatant isolated from the WT, *ΔtagH*, and *ΔtagHΔhlyA* strains against 1% rabbit red blood cells at 37°C for 5 h. LB was used as a negative control. The supernatant was collected every hour to measure absorbance at 540 nm. The relative hemolysis rates are shown as the means and standard errors of three biological replicates (n = 3). **c** Detection viability of SW480 cells using the CCK-8 assay. SW480 cells were treated with the WT, *ΔtagH*, and *ΔtagHΔhlyA* culture supernatants with 5%, 10%, and 15% dilutions at 37°C for 8 h. The values are shown as means and standard errors of five biological replicates (n = 5). ***, *P* < .001 between two strains. **d** Western blotting analysis for the precipitated culture supernatants and whole cell lysates from the WT, *ΔtagH, ΔtagH::tagH, ΔtagHΔhlyA, ΔtssB* and *ΔtssM* strains with anti-HlyA serum. A Coomassie blue stained gel was used to estimate the loading amount for each sample. All the experiments were independently repeated three times, and one representative result is shown.

As HlyA is the predominant hemolysin in *V. cholerae*, we tested whether TagH regulates *V. cholerae* hemolytic activity and if cytotoxicity depends on the expression of HlyA. We constructed the *ΔtagHΔhlyA* double knockout strain and performed hemolysis and cytotoxicity experiments. These results showed that the hemolytic activity of the *ΔtagHΔhlyA* culture supernatant was almost completely lost; it was significantly lower than both that of the *ΔtagH* mutant and wild-type (Fig. 1a, 2a, and 2b). In addition, the cytotoxicity of the *ΔtagHΔhlyA* culture supernatant to the SW480 cells was significantly lower than that of the *ΔtagH* mutant and wild-type (Figure 2c). Western blotting was then performed to detect the expression of HlyA in the culture supernatant and whole cell lysates. The results showed that HlyA expression in the supernatant and whole cell lysates of the *ΔtagH* mutant was significantly higher than in the wild-type strain, and HlyA expression was restored to the wild-type level in the complementary mutant *ΔtagH::tagH* (Figure 2d). There was no HlyA expression in the *ΔtagHΔhlyA* double knockout strain. Mass spectrometry analysis confirmed that the blot band in the western blotting analysis was the HlyA protein (Supplementary Fig. 1). Taken together, these data support the hypothesis that TagH regulates the hemolytic activity and cytotoxicity of *V. cholerae*, which depend on the expression of HlyA. We then aimed to determine whether the impact of TagH on HlyA expression is a downstream consequence of the impaired secretions from the T6SS. We constructed T6SS null mutants *ΔtssB* (*VCA0107*) and *ΔtssM* (*VCA0120*),^39^ and detected the expression of HlyA. The results showed that both the *tssB* and *tssM* knockouts resulted in the loss of Hcp secretion (Figure 1d) and slightly decreased hemolytic activity (Figure 1a) and HlyA expression (Figure 2d), suggesting that the regulation by TagH of the hemolytic activity of *V. cholerae* may not depend on the integrity of the T6SS.

### TagH negatively regulates HlyA transcription, and may involve the subtle regulation of hlyU and fur

**Figure 3.**
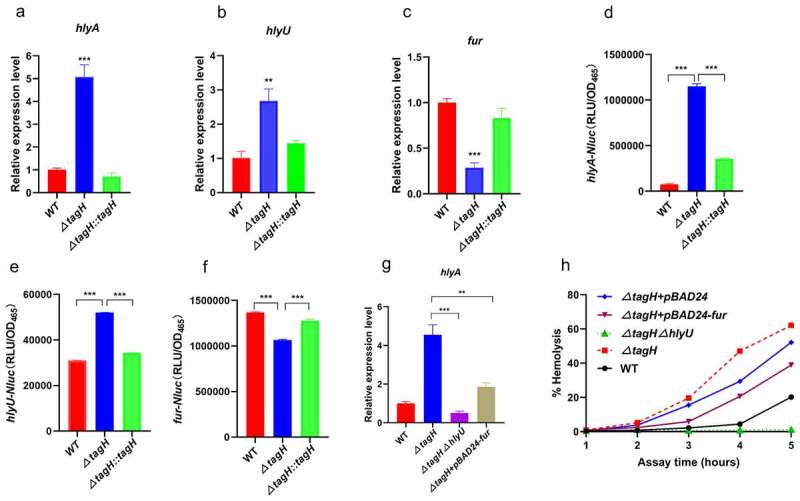
**Regulation of *hlyA* transcription by TagH may involve the subtle regulation of HlyU and Fur. a-c** qRT-PCR results for the relative mRNA levels of *hlyA* (a), *hlyU* (b), and *fur* (c) were compared between WT, *ΔtagH*, and *ΔtagH::tagH* strains. The values are shown as the means and standard errors of three biological replicates (n = 3). **, *P* < .01 and ***, *P* < .001 for the *ΔtagH* mutant relative to the wild-type strain. **d-f** The promoter activities were detected using a luciferase reporter assay. The luciferase activity of *hlyA, hlyU*, and *fur* were determined in the WT, *ΔtagH*, and *ΔtagH::tagH* strains. The values are shown as the means and standard errors of three biological replicates (n = 3). **g** qRT-PCR results for the relative mRNA levels of *hlyA* were compared between WT, *ΔtagH, ΔtagHΔhlyU*, and *ΔtagH+pBAD24-fur* strains. The values are shown as the means and standard errors of three biological replicates (n = 3). **, *P* < .01 and ***, *P* < .001 between two strains. **h** Hemolytic activity analysis of the culture supernatant isolated from the WT, *ΔtagH, ΔtagHΔhlyU, ΔtagH+pBAD24-fur*, and *ΔtagH+pBAD24* strains against 1% rabbit red blood cells at 37°C for 5 h.

Previous studies have suggested that the expression of HlyA in *V. cholerae* is mainly regulated at transcriptional and post-translational levels.^40,41^ At the transcriptional level, *hlyA* transcription is mainly regulated by HapR, Fur, and HlyU.^42^ The quorum-sensing regulator HapR inhibits HlyA expression at the transcriptional and post-transcriptional levels.^43^ The iron uptake regulator Fur can inhibit the expression of the *hlyA* gene,^44^ while the HlyU protein is a positive regulator of the *hlyA* gene.^45–47^ To investigate whether transcriptional regulation is involved, the expression of *hlyA* at the transcriptional level was assessed using quantitative real-time PCR (qRT-PCR). The results showed that the transcription level of the *hlyA* gene in the *ΔtagH* mutant was approximately five times higher than that of the wild-type, and in the *ΔtagH::tagH* strain it was restored to the wild-type level (Figure 3a). This suggests that TagH can regulate the expression of the *hlyA* gene at the transcriptional level. We further screened the expression of the above-mentioned *hlyA* transcriptional factors in the wild-type, *ΔtagH*, and *ΔtagH::tagH* strains using qRT-PCR. The results showed that the transcription level of *hlyU* in the *ΔtagH* mutant was significantly higher than that in the wild-type (Figure 3b), while the transcription level of *fur* in the *ΔtagH* mutant was significantly lower than that in the wild-type (Figure 3c). The transcription levels of *hlyU* and *fur* in *ΔtagH::tagH* were restored to levels similar to those in the wild-type. These results suggest that the transcriptional regulation of *hlyA* by TagH may be involved in HlyU and Fur. In addition, the luminescence reporter assay was used to measure transcription changes of *hlyA, hlyU*, and *fur* genes. The results showed that the activity of the *hlyA* promoter was significantly upregulated in the *ΔtagH* mutant when compared to the wild-type, while the activity of the *hlyA* promoter in the *ΔtagH::tagH* mutant was restored to a level close to that of the wild-type (Figure 3d), which is consistent with TagH repression of *hlyA* transcription. Additionally, the promoter activities of *hlyU* and *fur* were significantly upregulated and downregulated, respectively, in the *ΔtagH* mutant when compared to the wild-type (Figure 3e, 3f); this was consistent with the results of the qRT-PCR. We further constructed *ΔtagH+pBAD24-fur* strains to restore *fur* expression as well as *ΔtagHΔhlyU* double-knockout strains and analyzed their hemolytic activity and *hlyA* transcription. The results showed that the hemolytic activity and *hlyA* transcription of *ΔtagHΔhlyU* were significantly lower than those of the *ΔtagH* mutant and the wild-type, while the hemolytic activity and *hlyA* transcription of *ΔtagH+pBAD24-fur* were partially restored compared with those of the *ΔtagH* strain (Figure 3g, 3h). Taken together, these results suggest that the regulation of *hlyA* transcription by TagH may be involved in the subtle regulation of HlyU and Fur.

### TagH regulation of HlyA expression may require protease PrtV at the post-translational level

**Figure 4.**
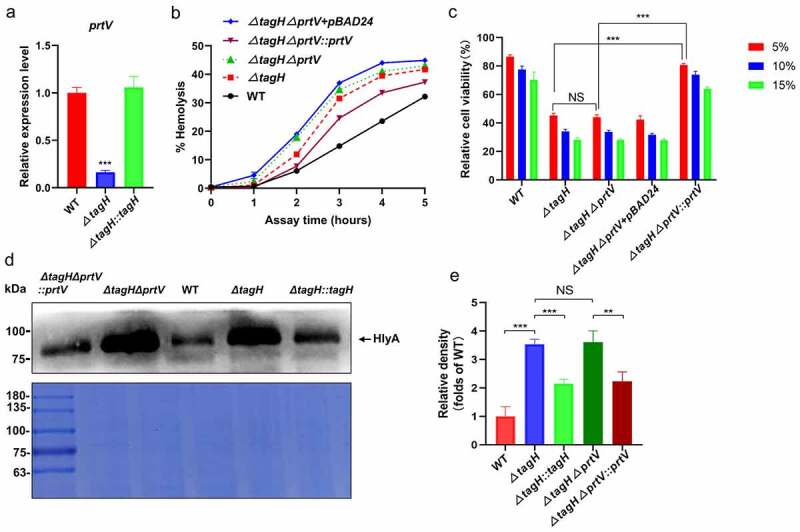
**PrtV may be involved in TagH regulation of HlyA expression at the post-translational level. a** qRT -PCR results for the relative mRNA levels of *prtV* were compared between the WT, *ΔtagH*, and *ΔtagH::tagH* strains. The values are shown as the means and standard errors of three biological replicates (n = 3). ***, *P* < .001 for the *ΔtagH* mutant relative to the wild-type strain. **b** Hemolytic activity assay showing the hemolytic activity of the culture supernatants collected from the WT, *ΔtagH, ΔtagHΔprtV, ΔtagHΔprtV-pBAD24* and the *prtV* complementary mutant *ΔtagHΔprtV::prtV* strains against 1% rabbit red blood cells at 37°C for 5 h. The supernatant was collected every hour to measure its absorbance at 540 nm. The relative hemolysis rates are shown as the means and standard errors of three biological replicates (n = 3). **c** Detection of SW480 cell viability using the CCK-8 assay. SW480 cells were treated with WT, *ΔtagH, ΔtagHΔprtV*, and *ΔtagHΔprtV::prtV* culture supernatants with 5%, 10%, and 15% dilutions at 37°C for 8 h. The values are shown as the means and standard errors of five biological replicates (n = 5). **d** Western blotting analysis of the precipitated supernatant proteins of the WT, *ΔtagH, ΔtagHΔprtV*, and *ΔtagHΔprtV::prtV* strains with anti-HlyA serum. The Coomassie-stained gel was used as a loading control. **e** The relative protein expression levels of HlyA were quantified according to the western blotting bands using ImageJ software. The values are shown as the means and standard errors of three biological replicates (n = 3). **, *P* < .01 and ***, *P* < .001; NS, not significant.

Evidence has been presented for the transcriptional regulation of *hlyA* by TagH. At the post-translational level, HlyA is produced as a precursor protein (pro-HlyA), which is subsequently activated by a protease conducting two cleavage steps to produce the fully active form of hemolysin.^40^ However, prolonged incubation of HlyA with protease can lead to the degradation of HlyA.^40,43^ In *V. cholerae*, HapR can increase the expression of the metalloproteases HapA and PrtV, which can degrade HlyA.^43^ In this study, we also found that the transcription level of *prtV* in the *ΔtagH* mutant was significantly downregulated when compared to the wild-type, and its transcription level in the *ΔtagH::tagH* mutant was restored to that of the wild-type (Figure 4a), suggesting that the *tagH* deletion inhibits the transcription of *prtV*. To further explore whether the effects of TagH on the activity of HlyA might require the protease PrtV, we constructed a *ΔtagHΔprtV* double knockout mutant, restored the expression of PrtV in the *ΔtagHΔprtV* mutant (*ΔtagHΔprtV::prtV*) using the pBAD24-*prtV* plasmid, then tested their hemolytic activity and cytotoxicity. The results showed that the hemolytic activity of the *ΔtagHΔprtV* mutant supernatant was slightly higher than that of the *ΔtagH* mutant (Figure 4b), but the cytotoxicity of the two strains did not change significantly (Figure 4c). However, both the hemolytic activity and cytotoxicity of the *ΔtagHΔprtV::prtV* strain were significantly lower than those of the *ΔtagH* and *ΔtagHΔprtV* strains (Figure 4b and 4c). This suggested that TagH’s regulation of hemolytic activity and cytotoxicity may also be mediated by its regulation of PrtV expression. We further tested whether PrtV affected the expression of HlyA in the supernatant of the above strains using western blotting. HlyA was expressed at a similar level in the supernatants of the *ΔtagH* and *ΔtagHΔprtV* strains (Figure 4d, 4e). However, when PrtV was restored in the *ΔtagHΔprtV* strain, the expression of HlyA was significantly decreased in *ΔtagHΔprtV::prtV* when compared to that in *ΔtagH* and *ΔtagHΔprtV* mutants. These results are consistent with the results of the tests for hemolytic activity and cytotoxicity in the *ΔtagH, ΔtagHΔprtV*, and *ΔtagHΔprtV::prtV* strains. Cumulatively, these data suggest that PrtV may be involved in TagH regulation of HlyA expression at the post-translational level.

### Putative phosphopeptide binding residues of TagH play a key role in regulating HlyA expression

**Figure 5.**
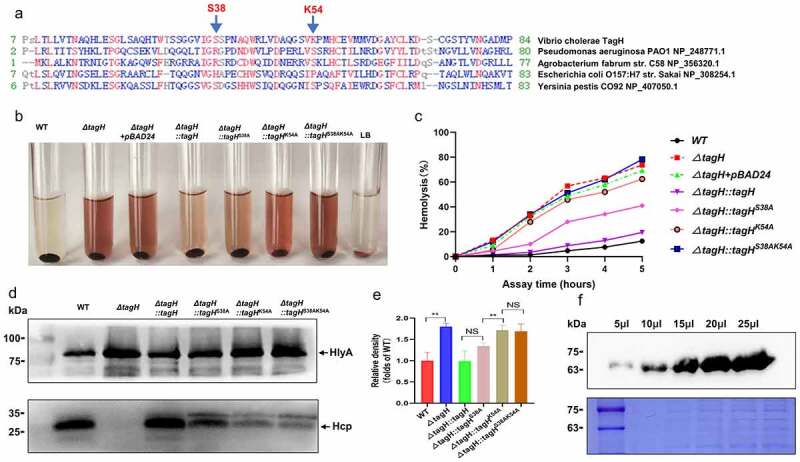
**Conserved phosphopeptide binding residues of TagH are required for the expression of HlyA and Hcp in culture supernatant. a** A partial sequence alignment of TagH with selected FHA domain family proteins. Conserved phosphopeptide binding residues are indicated with an arrow, and S38 and K54 were used for mutagenesis. **b-c** Hemolytic activity assay results showing the activity of the culture supernatant isolated from the WT, *ΔtagH, ΔtagH::tagH, ΔtagH::tagH*^S38A^, *ΔtagH::tagH*^K54A^, and *ΔtagH::tagH*^S38AK54A^ strains against 1% rabbit red blood cells at 37°C for 5 h. The supernatant was collected every hour to measure its absorbance at 540 nm. The relative hemolysis rates are shown as the means and standard errors of three biological replicates (n = 3). **d** Western blotting analysis of the precipitated supernatant proteins of the WT, *ΔtagH, ΔtagH::tagH, ΔtagH::tagH*^S38A^, *ΔtagH::tagH*^K54A^ and *ΔtagH::tagH*^S38AK54A^ strains with anti-HlyA and anti-Hcp serum. The Coomassie-stained gel was used as a loading control. All the experiments were independently repeated three times, and one representative result is shown. **e** Relative protein expression levels of HlyA quantified according to the western blotting bands using ImageJ software. The values are shown as the means and standard errors of three biological replicates (n = 3). **, *P* < .01; NS, not significant. **f** TagH phosphorylation analysis using Phos-tag SDS-PAGE. Different volumes of the His-tag-TagH protein in the *ΔtagH::tagH* mutant were separated on a 10% Phos-tag SDS-PAGE and examined using a specific anti-His-tag antibody. The Coomassie-stained gel was used as a loading control.

Structural studies have shown that several conserved FHA domain residues directly bind to the phosphate moieties and play key roles in their interactions with phosphorylated substrates.^33,48,49^ TagH sequence alignments with other FHA domain-containing proteins revealed that its FHA domain also contains conserved residues related to phosphopeptide binding (S38 and K54) (Figure 5a). To test whether these conserved phosphopeptide binding residues are also related to TagH’s regulation of the hemolytic activity in *V. cholerae*, we constructed various *tagH* mutants, including alanine substitution mutants at the Ser 38 residue (TagH^S38A^), Lys 54 residue (TagH^K54A^), and at both the Ser 38 and Lys 54 residues (TagH^S38AK54A^). The hemolytic activity was completely restored in the *ΔtagH::tagH* strain by the non-mutated pBAD24-*tagH* plasmid, whereas the hemolytic activity of the *ΔtagH::tagH*^S38A^, *ΔtagH::tagH*^K54A^, and *ΔtagH::tagH*^S38AK54A^ mutants increased successively, and they were all significantly higher than those of the complementary mutant *ΔtagH::tagH* (Figure 5b, 5c). This indicates that the putative phosphopeptide binding residues (S38 and K54) of TagH may be critical for the regulation of hemolytic activity in *V. cholerae*. Western blotting analysis also showed that the expression levels of HlyA in the supernatant of the *ΔtagH::tagH*^S38A^, *ΔtagH::tagH*^K54A^, and *ΔtagH::tagH*^S38AK54A^ strains increased successively. HlyA secretion was not restored in the Δ*tagH::tagH*^S38AK54A^ strain, whereas in *ΔtagH::tagH*^S38A^ and *ΔtagH::tagH*^K54A^ it was partially recovered when compared to that in *ΔtagH* (Figure 5d, 5e), which was consistent with the hemolysis phenotype. Additionally, we found that compared with the non-mutant, the S38A and K54A single-point mutants and the S38AK54A double-point mutant significantly inhibited the secretion of Hcp (Figure 5d). Taken together, these data support the hypothesis that the putative phosphopeptide-binding residues play a key role in regulating HlyA expression and Hcp secretion in *V. cholerae*. Since no conserved PpkA and PppA were found in *V. cholerae*, it is not clear if TagH can be phosphorylated. We used Phos-tag SDS-PAGE to separate phosphorylated and unphosphorylated proteins on a gel, and western blotting was performed using an anti-His-tag antibody. The results showed that there was only one TagH protein band (Figure 5f). We attempted to further identify potential phosphorylation sites in TagH by mass spectrometry, but no sites were identified (Supplementary Table 1). These data suggest that there is no phosphorylated phenotype of TagH.

### tagH mutants enhance the enterotoxicity of V. cholerae in rabbit ileal loops

**Figure 6.**
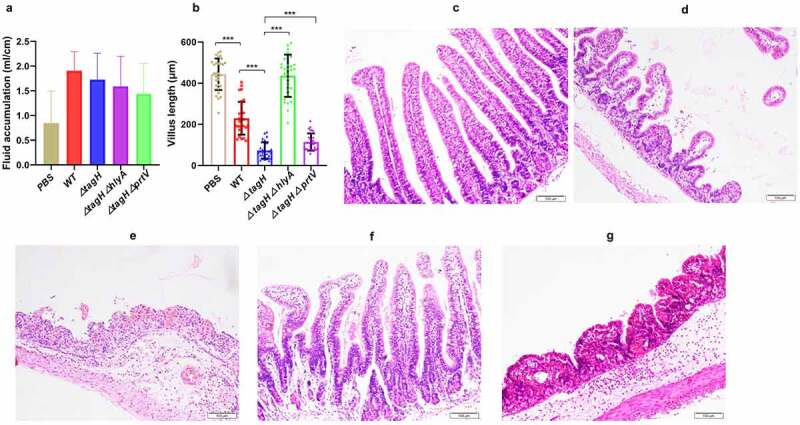
**Deletion of *tagH* enhances the enterotoxicity of *V. cholerae*, which depends on HlyA. a** The loop fluid volume (mL)/length (cm) ratio of rabbit after infection with 1 × 10^8^ CFU of the WT, *ΔtagH, ΔtagHΔhlyA*, and *ΔtagHΔprtV* strains, n = 5. **b** Intestinal villus length was measured using ImageJ software, n = 30. **c-g** Histological damage in the rabbit ileal loop treated for 18 h with 1 × 10^8^ CFU of the WT (d), *ΔtagH* (e), *ΔtagHΔhlyA* (f), and *ΔtagHΔprtV* (g) strains (H&E staining, 100 ×). PBS (c) was used as a negative control. ***, *P* < .001 between two strains. All scale bars = 100 µm.

To investigate the contributions of TagH, HlyA, and PrtV to the enterotoxicity of *V. cholerae*, we examined the enterotoxicity of the wild-type, *ΔtagH, ΔtagHΔhlyA*, and *ΔtagHΔprtV* strains in rabbit ileal loops. After 18 h of infection, the fluid accumulation (FA) in each loop was measured. The FA ratios for the wild-type, *ΔtagH, ΔtagHΔhlyA*, and *ΔtagHΔprtV* strains were not significantly different (Figure 6a). Histological analysis showed that when compared with the phosphate-buffered saline (PBS) control (Figure 6c), the rabbit ileal loops treated with the wild-type strain showed shorter villus length (Figure 6b) and crypt depth, hyperemia, and neutrophil infiltration in the submucosa (Figure 6d). Sections from tissues infected with the *ΔtagH* mutant showed more severe diffuse necrotizing enteritis, characterized by an almost complete loss of the villous structure, denudation of the surface epithelium in some sections, massive neutrophil infiltration in the submucosa, and crypt hyperemia and expansion (Figure 6e). Rabbit ileal loops treated with the *ΔtagHΔhlyA* mutant showed no significant intestinal histopathological changes (Figure 6f), and the villus lengths were not significantly different from those of the PBS group (Figure 6b). The ileal loops treated with *ΔtagHΔprtV* had slightly weaker intestinal pathological changes and longer villous lengths when compared with the *ΔtagH* mutants infected group (Figure 6b, 6g). Taken together, these results strongly suggest that the *tagH* deletion mutant enhances the enterotoxicity of *V. cholerae*, which mainly depends on HlyA, and that PrtV has a weaker effect.

### tagH mutants enhance the extraintestinal pathogenicity of V. cholerae dependent on HlyA in the sepsis model

**Figure 7.**
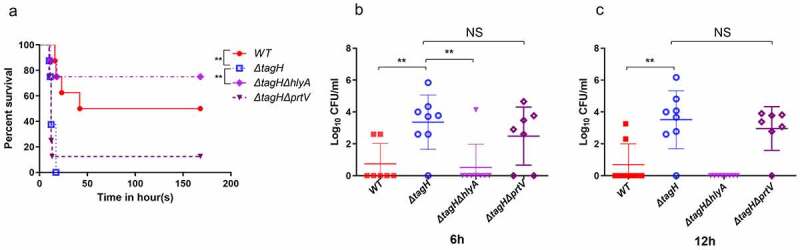
**HlyA is the main contributor to the enhanced invasiveness of *ΔtagH* mutants. a** The survival rates of mice after infection with 1 × 10^7^ CFU of the WT, *ΔtagH, ΔtagHΔhlyA*, and *ΔtagHΔprtV* strains, n = 8. **b-c** Bacteria numbers in the blood samples of mice after infection with 1 × 10^7^ CFU of the WT, *ΔtagH, ΔtagHΔhlyA*, and *ΔtagHΔprtV* strains for 6 h (b) and 12 h (c). The data are expressed as the mean log10 CFUs of per mL blood samples (± SD, n = 8). **, *P* < .01 and ***, *P* < .001; NS, not significant.

In this study, a sepsis model was established by intraperitoneal infection of *V. cholerae* to determine whether the *tagH* mutant affects the extraintestinal pathogenicity of the non-O1/non-O139 *V. cholerae*. The results showed that the survival rates of mice infected with the wild-type, *ΔtagH, ΔtagHΔhlyA*, and *ΔtagHΔprtV* strains were 50%, 0%, 75%, and 12.5%, respectively (Figure 7a). Surprisingly, all mice in the *ΔtagH* infection group died within 17 h, but in the *ΔtagHΔhlyA* infection group only one mouse died within 17 h. These results strongly suggest that the *tagH* deletion mutant enhances the extraintestinal pathogenicity of *V. cholerae*, which depends on HlyA. There was no significant difference in the average survival time and survival rate of the mice infected with the *ΔtagHΔprtV* and *ΔtagH* strains, suggesting that the effect of the PrtV is not significant in the extraintestinal pathogenicity of non-O1/non-O139 *V. cholerae*. Mouse blood samples were collected at 6 h and 12 h after bacterial infection for bacterial load analysis. The blood bacterial load of the mice infected with the *ΔtagH* strain was significantly higher than that of the wild-type (Figure 7b, 7c). Only one mouse in the *ΔtagHΔhlyA* infection group was found to have bacteria in its blood at 6 h. The average bacterial load in the blood of mice infected with the *ΔtagHΔprtV* strain was not significantly different from that of the *ΔtagH* infection group. These data provide further evidence that HlyA is the main contributor to the enhanced extraintestinal invasiveness of the *ΔtagH* mutants.

**Figure 8.**
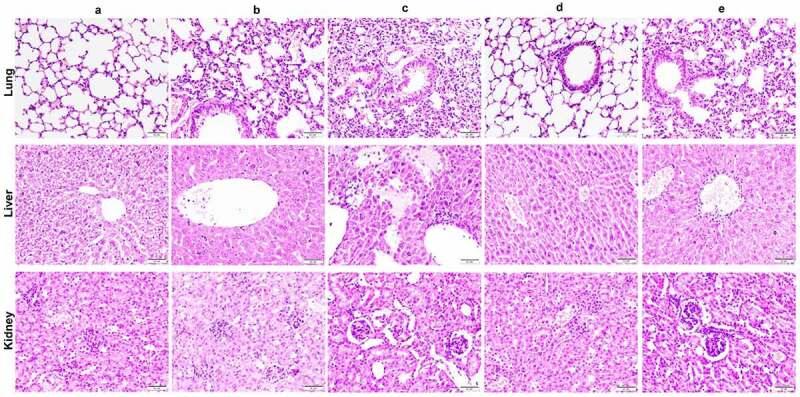
**Deleting *tagH* enhances the extraintestinal pathogenicity of *V. cholerae*, which depends on HlyA**. Hematoxylin and eosin stained sections of mice lungs, livers, and kidneys challenged after infection with 1 × 10^7^ CFU of WT (b), *ΔtagH* (c), *ΔtagHΔhlyA* (d), and *ΔtagHΔprtV* (e) strains for 12 h. The PBS (a) treatment group was used as a negative control. Sections were photographed at 200 × magnification. All scale bars = 50 µm.

Histological analysis showed that when compared with the PBS control group (Figure 8a), the lung tissue of the wild-type infection group had thickened alveolar walls, infiltration of inflammatory cells into the alveolar space, interstitial edema, and lung congestion (Figure 8b). When compared with the wild-type infection group, the *ΔtagH* infected mice had significantly increased lung injury, characterized by more alveolar wall congestion and septal widening, as well as increased neutrophil infiltration (Figure 8c). In the *ΔtagHΔhlyA* infected mice (Figure 8d), only a small number of inflammatory cells were observed in the bronchial tube pulmonary alveoli. Pathological damage in the lungs of the *ΔtagHΔprtV* infected mice (Figure 8e) was slightly weaker than that in the *ΔtagH* infected mice. In addition, we found that the liver tissue of the mice infected with the *ΔtagH* mutant had the highest infiltration of inflammatory cells when compared to the other groups, and focal necrosis of the liver cells was observed (Figure 8c). The infiltration of inflammatory cells in the liver tissue of the *ΔtagHΔprtV* mutant infected group (Figure 8e) was slightly higher that of the wild-type group (Figure 8b), while no obvious inflammatory change was observed in the *ΔtagHΔhlyA* infected group (Figure 8d). Notably, mice infected with *ΔtagH* and *ΔtagHΔprtV* mutants showed extensive atrophy of glomerular capillary loops and mild proliferation of mesangial stroma and mesangial cells (Figure 8c and 8e). WT and *ΔtagHΔhlyA* infected groups showed no obvious pathological changes (Figure 8b and 8d). Collectively, these results suggest that the *ΔtagH* mutant can significantly enhance the extraintestinal invasion ability and pathogenicity of *V. cholerae*, and HlyA is the main contributor to this process.

## Discussion

In this study, we identified for the first time that TagH plays an important role in controlling *V. cholerae* hemolytic activity and virulence, in addition to regulating the T6SS. Previous studies have shown that Thr phosphorylation events in the FHA domain protein regulate its binding to phosphorylated substrates in *P. aeruginosa*^33,50^ and *S. marcescens*,^51^ which has a key role in the T6SSs assembly and secretions. However, while *Agrobacterium tumefaciens* can encode PpkA and PppA, the phosphorylation target of its PpkA is not Fha but TssL.^49^ T6SS activation and secretion requires the interaction of the pThr-binding motif of Fha and phosphorylated TssL. In this study, we also confirmed that *tagH* knockout can inhibit Hcp secretion, which is consistent with previous reports.^37^ Interestingly, unlike T6SS null mutants *ΔtssB* and *ΔtssM*, we found that *tagH* knockout can significantly increase intracellular Hcp expression (Figure 1d), which may be due to the regulation of Hcp expression by HlyU. Previous studies reported that the expression of both HlyA and Hcp proteins depended on HlyU.^52^ In *V. cholerae* it is currently unknown if, due to the absence of PpkA, TagH can be phosphorylated, and we were unable to detect any phosphorylation in TagH (Figure 5f and Supplementary Table 1). However, we found that the conserved phosphopeptide binding sites (S38 and K54) of TagH were critical for HlyA expression and T6SS secretion. These results suggest that the binding of the FHA phosphopeptide domain to the phosphorylated substrate may be more conserved and extensive than its own phosphorylation events among different bacteria. Notably, the *ΔtagH::tagH*^K54A^ mutant had a stronger effect on HlyA expression and Hcp secretion than *ΔtagH::tagH*^S38A^. Similar results were found in *Pseudomonas aeruginosa*, showing that the Fha1^S48A^ mutation (corresponding to TagH^K54A^) causes more Hcp secretion defects than the Fha1^R32A^ mutation (corresponding to TagH^S38A^).^33^ These results suggest that the conserved phosphopeptide binding residue K54 may have a more important role than the S38 residue in the binding of phosphorylated substrates. Although our data showed that TagH could regulate the transcription of genes such as *hlyA, hlyU, fur*, and *prtV*, no typical DNA-binding domains were found in the TagH sequence. However, its conserved phosphopeptide binding sites play a critical role in the regulation of HlyA expression. Therefore, we speculate that TagH regulation of the hemolytic activity of *V. cholerae* may be indirect, and it may depend on the binding of the FHA domain to the phosphorylated substrate.

A review of previously published data showed that HlyA is strictly controlled by multiple regulatory factors at the transcriptional and post-translational levels.^40,42,43^ At the transcriptional level, our results showed that the deletion of *tagH* significantly upregulated the transcription and promoter activity of *hlyA* and *hlyU* and downregulated *fur* transcription (Figure 3). The binding site of HlyU in the promoter region of *hlyA* is located at −563···−627, while the binding site of Fur in the promoter region of *hlyA* is located at −545··· −596^42^, indicating that two binding sites partially overlap. Therefore, we speculate that the transcriptional regulation of *hlyA* by TagH may depend on the subtle coordination of HlyU and Fur, which is consistent with a previous report.^42^ Furthermore, a previous study found that HlyA was highly expressed in the early stages of logarithmic growth,^42^ and our research mainly focused on this stage. At the post-translational level, several proteases have been shown to be involved in the activation and degradation of HlyA in *V. cholerae*.^40^ *Vibrio mimicus* has also been shown to display a very similar phenomenon, in which endogenous metalloproteases cleave and activate hemolysin at an early stage but inactivate hemolysin after further incubation.^53^ PrtV of *V. cholerae* is a Zn^2+^-binding extracellular protease belonging to the M6 metalloprotease family.^54,55^ HlyA is a substrate of the PrtV protease, and PrtV is the main contributing factor to HlyA inactivation.^56^ Our data showed that the *tagH* knockout significantly inhibited the transcription of the *prtV* gene (Figure 4a), and the *ΔtagHΔprtV* mutant only slightly increased HlyA expression when compared to the *ΔtagH* mutant (Figure 4d). We speculate that the *tagH* knockout inhibits *prtV* transcription at a very low level (approximately 16% of the wild level), resulting in no further impact on HlyA expression when *prtV* is knocked out in the context of the *ΔtagH* mutants.

Although purified HlyA has been reported to induce fluid accumulation (FA), it was also found that the amount of FA varies in the different parts of the intestine.^57^ In addition, during *V. cholerae* infection, other virulence factors, such as CT-like enterotoxin, can induce FA production, which interferes with the amount of FA induced by HlyA.^58^ Therefore, the amount of FA may not be a good indicator of the intestinal toxicity of *V. cholerae*. A previous study also found that there was no significant difference in the amount of FA between the *V. cholerae* El Tor biotype and its *hlyA* knockout mutants, but there was a significant difference in the degree of pathological damage.^56^ In this study, the rabbit ileal loop infection experiment results showed that the *tagH* knockout could enhance *V. cholerae* damage to the intestinal tissue, and this process mainly depended on HlyA (Figure 6), as the *ΔtagHΔhlyA* strain did not cause intestinal injury. Previous studies have also shown that the deletion of the *hlyA* gene results in a 100 times reduction in the virulence of *V. cholerae* in mice.^21^ Indeed, HlyA has been described as a cause of tissue damage in the small intestinal epithelium of adult mouse infection models.^59^ The pathogenicity of the *ΔtagHΔprtV* strain was slightly less than that of the *ΔtagH* strain in the rabbit loop and septicemia models (Figure 6 and Figure 7), suggesting that PrtV also plays a small role in the pathogenic process. Cases of non-O1/non-O139 *V. cholerae* sepsis are gradually increasing and the mortality rate is approximately 33%,^60^ but the main contributing factors to its pathogenesis remain unclear. We found that when compared with the wild-type strain, the *ΔtagH* mutant was more likely to invade the bloodstream, while the *ΔtagHΔhlyA* strain rarely entered the bloodstream. (Figure 7b and 7c). In addition, the survival rate of mice infected with the *ΔtagH* mutant was significantly lower than that of mice infected with the wild-type and *ΔtagHΔhlyA* strains (Figure 7a). Therefore, our results suggest that HlyA is a key contributing factor in non-O1/non-O139 *V. cholerae* sepsis. Hemolysin is also considered to be the main contributor to hemorrhagic septicemia, a characteristic of vibriosis.^61^ We speculate that HlyA can disrupt cell connections, helping bacteria break through the barrier and enter the bloodstream, and leading to disseminated infections. Furthermore, Ou et al. found that HlyA can cause epithelial cell death at high concentrations, while at low concentrations, it can reduce the stability of tight cell junctions.^56^

**Figure 9.**
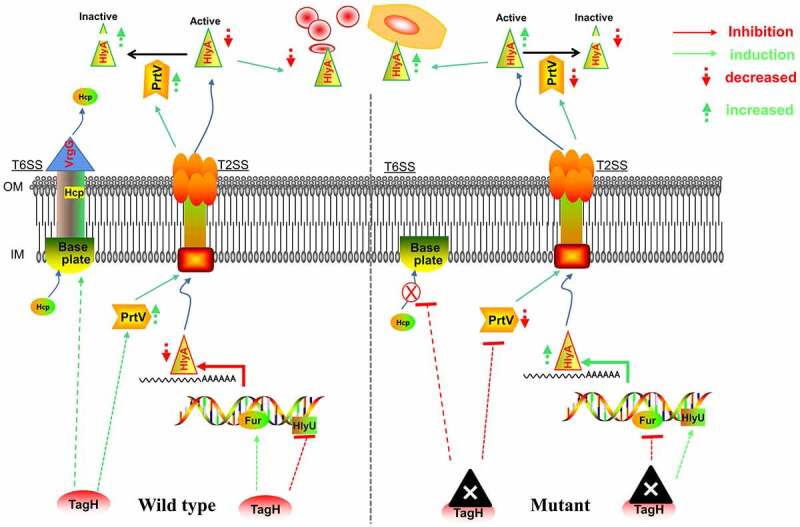
**A proposed model showing how the secretion of HlyA and Hcp are negatively and positively regulated by TagH in *V. cholerae*, respectively**. In the normal state (wild-type), TagH promotes T6SS assembly and Hcp secretion, inhibits HlyA transcription through the synergistic effects of HlyU and Fur, and promotes HlyA degradation by PrtV protease. In the *tagH* knockout state (mutant), the assembly of the T6SS and Hcp secretion were inhibited. The transcription of *hlyA* was upregulated through the subtle regulation of HlyU and Fur; and the transcription of the *prtV* gene was downregulated, resulting in the decreased degradation of HlyA. The red arrow represents inhibition and the downward dashed red arrow represents a decrease; The green arrow indicates induction and the upward green dashed arrow represents an increase. IM, inner membrane; OM, outer membrane.

Taken together, we propose a hypothetical model in which the secretion of HlyA and the activity of the T6SS is negatively and positively regulated by TagH in *V. cholerae*, respectively (Figure 9). This study provides evidence to help unravel the regulatory role of TagH in HlyA and the T6SS, expands our understanding of the function of TagH and HlyA, and provides critical insights for the development of strategies to manage HlyA, which plays a key role in the invasion and pathogenesis of *V. cholerae*. However, this study had some limitations. The intermediate bridge molecule TagH plays a regulatory role that has not yet been identified, and some regulatory details have not been fully elucidated. Future research is required to address the details of the regulation of TagH in the physiological and pathogenic processes of *V. cholerae*.

## Methods

### Bacterial strains and growth conditions

The non-O1/non-O139 *V. cholerae* HN375 strain was obtained from the China Center for Type Culture Collection (accession number CCTCC AB209168),^62^ and was used as the wild-type strain in this study. *E. coli* DH5α, DH5αλpir, BL21(DE3), and WM3064 were used for cloning, expression, and as the donor strains in the conjugative transfer experiments. All strains were cultured in LB with shaking or LB agar at 37°C. The following concentrations of antibiotics: 50 µg/mL kanamycin, 100 µg/mL ampicillin, and 50 µg/mL gentamycin were added to the culture medium for *E. coli* and *V. cholerae* when necessary. All bacterial strains and plasmids used in this study are listed in Supplementary Table 2.

### DNA manipulations and genetic techniques

All in-frame deletion mutants were constructed from the wild-type HN375 strain using the suicide plasmid pWM91, as described previously.^63^ All primers used are listed in Supplementary Table 3. To construct the complementary mutants, the entire coding regions of *tagH* (*VCA0112*) and *prtV* (*VCA0223*) were cloned into the pBAD24 vector,^64^ and then conjugated into *V. cholerae ΔtagH* and *ΔtagHΔprtV* mutants (designated as *ΔtagH::tagH* and *ΔtagHΔprtV::prtV*, respectively). In order to supplement the expression of *fur* in *ΔtagH* strain, the entire coding region of *fur* (*VC2106*) was cloned into the pBAD24 vector, and then conjugated into *V. cholerae ΔtagH* mutant (designated as *ΔtagH+pBAD24-fur*). We constructed the S38A, K54A, and S38AK54A point mutations of *tagH* using site-directed mutagenesis, as was previously described,^65^ and the templates used were pBAD24-*tagH* plasmids. The pBAD24-*tagH*^S38AK54A^, pBAD24-*tagH*^S38A^, and pBAD24-*tagH*^K54A^ vectors were conjugated to the *V. cholerae ΔtagH* mutant (designated as *ΔtagH::tagH*^S38A^, *ΔtagH::tagH*^K54A^, and *ΔtagH::tagH*^S38AK54A^, respectively). All complementary strains were cultured in LB medium supplemented with 0.1% arabinose to induce gene expression.

### Recombinant protein expression, purification, and preparation of polyclonal antisera

The coding regions of *hcp* (*VC1415*) and *hlyA* (*VCA0223*) were amplified and cloned into the plasmids pET28a and pCold-TF (Takara), respectively. These recombinant plasmids were transferred into *E. coli* BL21(DE3) strain for protein expression. The His-tagged recombinant proteins were purified using Ni^2+^-NTA affinity chromatography, as was previously described.^66^ To prepare anti-Hcp and anti-HlyA antiserum for Western blotting analysis, 6-week-old Kunming female mice (Experimental Animal Center, Zunyi Medical University) were randomly assigned to each group (n = 10) and raised in a specific pathogen-free (SPF) environment. Mice were immunized subcutaneously with 30 µg of recombinant Hcp and HlyA proteins with an equal volume of aluminum adjuvant on days 1, 14, and 28. Blood samples were collected from the hearts on day 42, and the serum titers were tested by ELISA.

### Western blot analysis

All experimental strains were cultured in LB medium or LB with 0.1% arabinose at 37°C with shaking at 220 rpm until the OD_600_ reached 0.6. Culture supernatants were separated by centrifugation. Secreted proteins were precipitated from 2 mL of culture supernatant using trichloroacetic acid-acetone precipitation, as described previously.^67^ The conventional samples were separated by SDS-PAGE, and the phosphorylation samples were separated by Phos-tag SDS-PAGE. The Phos-tag SDS-PAGE analysis was carried out according to the Phos-tag acrylamide user manual (Apexbio Technology LLC, USA) with a few modifications. The protein samples were separated on 10% polyacrylamide gel containing 0.375 M Tris-HCl (pH 8.8), 20 µM Phosbind, and 40 µM MnCl_2_, with electrophoresis conducted at 25–30 mA/gel under a maximum voltage of 120 V. After electrophoresis, the gel was immersed in a transfer buffer containing 10 mM EDTA and gently shaken for at least 10 min. Next, the gel was soaked in an EDTA-free transfer buffer and gently shaken for 10 min. The gel was then washed with transfer buffer containing 1% SDS for 15 min and transferred to PVDF membrane. Immunoblotting was performed with a 1:1000 dilution of anti-Hcp or anti-HlyA (Self-made antibody as described above) or anti-His-tag polyclonal antiserum (Proteintech Group, Inc) as the primary antibody. The HRP-conjugated goat anti-mouse antibody was prepared in a 1:5000 dilution as a secondary antibody. Duplicate gels were run and stained with Coomassie Brilliant Blue to confirm equal loading of the samples. All experiments were repeated thrice for each group.

### Quantitative real-time PCR (qRT-PCR)

The wild-type, *ΔtagH*, and *ΔtagH::tagH* strains were grown to a mid-exponential phase (OD_600_ of ~0.6) in LB liquid medium. Cells were pelleted by centrifugation for 2 min at 8000 × g. The total RNA was isolated using the TRIzol Reagent for cDNA synthesis. qRT-PCR assays were performed as was previously described.^68^ All experiments were repeated thrice for each group.

### Hemolytic activity assay

For the blood agar hemolysis test, experimental strains were inoculated on blood agar plates, incubated at 37°C for 24 h, and the diameter of the hemolysis loop was observed. For the culture supernatant hemolysis test, culture supernatants were collected at OD_600 _~0.6, and filter sterilized using 0.22 µm filters. A 10% fresh rabbit erythrocyte suspension was prepared by washing and diluting with saline. The culture supernatants and erythrocyte suspensions were mixed at a ratio of 9:1 and incubated at 37°C for 5 h. The supernatant was collected every hour to measure its absorbance at 540 nm. Trion-X (1%) and LB medium were used as positive and negative controls, respectively. The relative hemolysis rate was calculated based on 100% hemolysis with the Triton X-100 control. All experiments were repeated thrice for each group.

### Cytotoxicity assay

Cytotoxicity was determined using a CCK-8 assay as described previously.^69^ SW480 cells in the exponential growth phase were collected and seeded in 96-well plates at a density of 5 × 10^5^ cells per well for 24 h. Culture supernatants of the experimental strains were collected at OD_600 _~0.6 and filter sterilized using 0.22 µm. SW480 cells were treated with culture supernatants diluted at 5%, 10%, and 15%, and then cultured at 37°C. LB was used as a negative control. After 8 h, the cells were washed three times with PBS, the medium was replaced with FBS-free medium, then 10 µL of the CCK-8 solution was added, and the cells were incubated at 37°C for 3 h. The absorbance at 450 nm was determined for each well. The relative cell viability was calculated based on the 100% cell viability of the LB medium control. All experiments were repeated thrice for each group.

### Luciferase activity analysis

For the luciferase reporter analysis, we modified the pHRP309 plasmid^70^ and replaced the LacZ reporter gene with the Nluc reporter gene. Putative promoter regions were PCR-amplified and cloned upstream of the Nluc reporter gene. These plasmids were conjugated into the WT, *ΔtagH*, and *ΔtagH::tagH* strains, respectively. The strains were then cultivated in LB medium supplemented with 50 µg/mL gentamicin at 37°C with shaking at 220 rpm until the OD_600_ reached ~0.6. Cell pellets were collected, washed twice with ice-cold PBS, and resuspended in an equal volume of cold PBS. Cells were disrupted using ultrasound, and the resulting cell supernatants were detected using a Nano-Glo luciferase assay kit (Promega). All experiments were repeated three times for each group.

### Rabbit ileal loop infection assay

The rabbit ileal loop test was performed as described previously.^71^ Five New Zealand white rabbits for each group were adaptively fed with sterile water containing 1 mg/1 mL streptomycin for 72 h and fasted for 48 h before surgery with only water available *ad libitum*. Rabbits were anesthetized by intramuscular injection of tiletamine and zolazepam (0.1 mL/kg body weight) and xylazine hydrochloride injection (0.05 mL/kg body weight). After a celiotomy was performed, the ileal was washed using sterile water and ligated into discrete loops of approximately 6 cm using a sterile surgical line. Each loop was separated into uninoculated segments that were 1–2 cm. Each loop was injected with 1 × 10^8^ CFU of the experimental strain. PBS was used as a negative control. After approximately 18 h, the rabbits were sacrificed using sodium pentobarbital (150 mg/kg body weight), and the peritoneal cavity was opened to remove the loops. The loop fluid volume (mL)/length (cm) ratio was calculated to assess the extent of the fluid accumulation. To further investigate whether these strains caused tissue damage, the tissue from each intestinal test segment was collected, embedded, sectioned, and stained with H&E. Pathological images were acquired with Olympus BX53 microscope containing the cellSens System. The intestinal villus length was then measured using ImageJ software.

### Mouse septicemia infection model

Female CD1 mice (6–8 weeks old) were used to construct sepsis models. Mice were randomly assigned to each group (n = 8) and raised in a specific pathogen-free (SPF) environment. Each mouse was intraperitoneally injected with 1 × 10^7^ CFU/100 µL of each experimental strain. Negative control mice were injected with 100 µL of PBS only. Food was then immediately offered to all animals. For the blood cultures, the blood of the mice was collected 6 h and 12 h after infection. After serial dilution, 50 µL of the blood diluent was plated on LB agar and incubated at 37°C for 24 h. The number of colonies on the plates was then calculated. For histopathological observation, mice were sacrificed 12 h after the intraperitoneal injection by cervical dislocation under anesthesia (isoflurane inhalation). The liver, lung, and kidney tissues of the mice were subjected to pathological examination. Pathological images were acquired using the Olympus BX53 microscope containing the cellSens System. For survival experiments, mice were observed for 7 days after the intraperitoneal injection, and the death and survival times were recorded.

## Supplementary Material

Supplemental Material

## Data Availability

The data that support the findings of this study are available in “figshare” at a pre-registered DOI (http://10.6084/m9.figshare.16912660) and the reviewer URL https://figshare.com/s/571cc7fa349472c33f3d.

## Statistical analysis

Statistical analyses were performed using Prism (version 8) software (GraphPad Software, USA). The differences between two groups were analyzed using unpaired two-tailed Student’s t-test. Survival data were analyzed using the log-rank (Mantel-Cox) test. *P* < .05 was considered significant. All experiments were performed on at least three independent replicates, and the values are expressed as the mean ± SD.
