# Supplementary material for: Type VI secretion system-associated FHA domain protein TagH regulates the hemolytic activity and virulence of Vibrio cholerae
Source: Gut Microbes. 2022 Apr 6;14(1):2055440. doi: 10.1080/19490976.2022.2055440 (PMC8993066; doi:10.1080/19490976.2022.2055440)
Supplement: Supplemental Material [file KGMI_A_2055440_SM4978.zip › Supplementary information.docx]

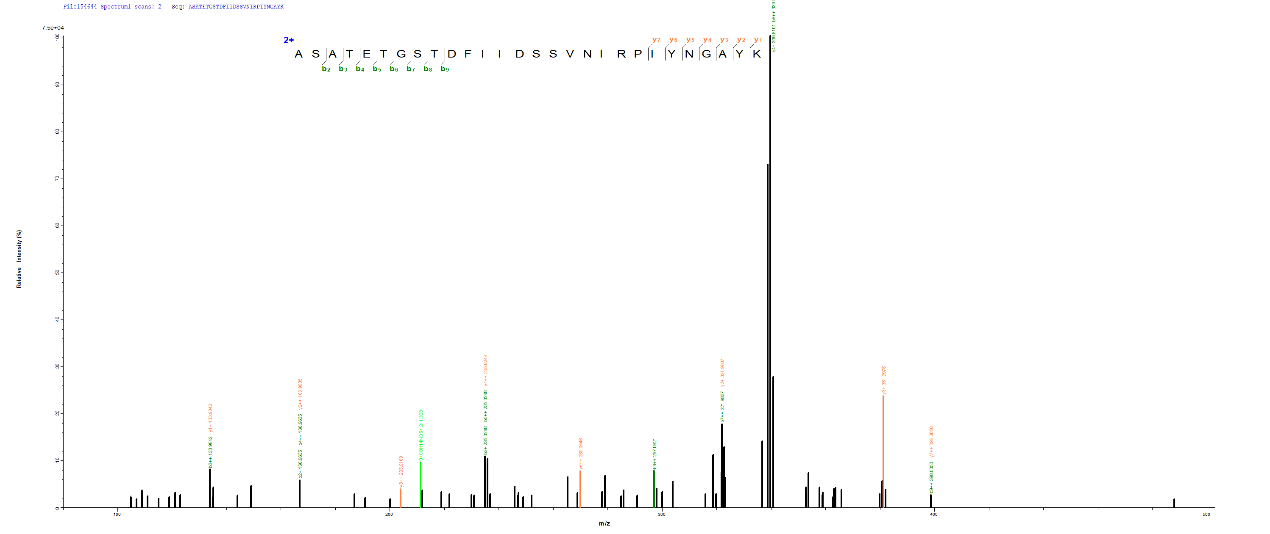


**Supplementary Fig.1 Mass spectrometry identification of HlyA.** According to the position corresponding to HlyA in Western blotting, the HlyA protein band in Coomassie blue staining duplicate gel was excised for mass spectrometry (MS) identification after in-gel digestion. MS/MS ion spectrum with the matched b and y ions of the peptide ASATETGSTDFIIDSSVNIRPIYNGAYK in HlyA is shown.

**Supplementary Table 1. Mass spectrometry identification of TagH phosphorylation.**

(**See Excel**)

**Supplementary Table 2. Bacterial strains and plasmids used in this study**

| **Strain or plasmid** | **Genotype or feature(s)** | **Reference or source** |
| --- | --- | --- |
| **Strains** |  |  |
| Non-O1/non-O139 *Vibrio cholerae* Strains |  |  |
| HN375 | Wild-type non-O1/non-O139 *V. cholerae* | Lab collections |
| *ΔtssB* | in-frame deletion of *tssB* | This study |
| *ΔtssM* | in-frame deletion of *tssM* | This study |
| *ΔtagH* | in-frame deletion of *tagH* | This study |
| *ΔtagH::tagH* | *ΔtagH* complemented with pBAD24-*tagH*, Amp^r^ | This study |
| *ΔtagHΔhlyU* | in-frame deletion of *tagH* and *hlyU* | This study |
| *ΔtagH+fur* | *ΔtagH* complemented with pBAD24-*fur*, Amp^r^ | This study |
| *ΔtagHΔprtV* | in-frame deletion of *tagH* and *prtV* | This study |
| *ΔtagHΔprtV::prtV* | *ΔtagHΔprtV* complemented with pBAD24-*prtV*, Amp^r^ | This study |
| *ΔtagHΔhlyA* | in-frame deletion of *tagH* and *hlyA* | This study |
| *ΔtagH::tagH*^S38A^ | *ΔtagH* complemented with pBAD24-*tagH*^S38A^, Am^r^ | This study |
| *ΔtagH::tagH*^K54A^ | *ΔtagH* complemented with pBAD24-*tagH*^K54A^, Am^r^ | This study |
| *ΔtagH::tagH*^S38AK54A^ | *ΔtagH* complemented with pBAD24-*tagH*^S38AK54A^, Amp^r^ | This study |
| *hlyA-nluc-*HN375 | HN375 complemented with pHRP309-*nluc*-*hlyA*, Gm^r^ | This study |
| *hlyA-nluc-ΔtagH* | *ΔtagH* complemented with pHRP309-*nluc*-*hlyA*, Gm^r^ | This study |
| *hlyA-nluc-ΔtagH::tagH* | *ΔtagH::tagH* complemented with pHRP309-*nluc*-*hlyA*, Gm^r^ | This study |
| *hlyU-nluc-*HN375 | HN375 complemented with pHRP309-*nluc*-*hlyU*, Gm^r^ | This study |
| *hlyU-nluc-ΔtagH* | *ΔtagH* complemented with pHRP309-*nluc-hlyU*, Gm^r^ | This study |
| *hlyU-nluc-ΔtagH::tagH* | *ΔtagH::tagH* complemented with pHRP309-*nluc-hlyU*, Gm^r^ | This study |
| *fur-nluc-*HN375 | HN375 complemented with pHRP309-*nluc-fur*, Gm^r^ | This study |
| *fur-nluc-ΔtagH* | *ΔtagH* complemented with pHRP309-*nluc-fur*, Gm^r^ | This study |
| *fur-nluc-ΔtagH::tagH* | *ΔtagH::tagH* complemented with pHRP309-*nluc-fur*, Gm^r^ | This study |
| ***E. coli* strains** |  |  |
| DH5α | F−, ø80dlacZΔM15, Δ(lacZYA-argF) U169 deoR, recA1, endA1, hsdR17 (rk-,mk+), phoA, supE44, ʎ-, thi-1, gyrA96, relA1 | Lab collections |
| DH5α( λpir) | supE44 ΔlacU169 (ΦlacZΔM15) recA1 endA1 hsdR17 thi-1 gyrA96 relA1 λpir | Lab collections |
| WM3064 | thrB1004 pro thi rpsL hsdS lacZΔM15 RP4-1360 Δ(araBAD)567 ΔdapA1341::[erm pir] | Lab collections |
| BL21 | F-, ompT, hsdSB (rB-mB-), gal, dcm | Lab collections |
| BL21（DE3） | F-, lon-11, Δ(ompT-nfrA)885, Δ(galM-ybhJ)884, λDE3[lacI lacUV5-T7 gene 1 ind1 sam7 nin5], Δ46, [mal+]K-12(λS), hsdS10 | Lab collections |
| pCold TF-*hlyA*-BL21 | BL21 complemented with pCold TF-*hlyA*, Amp^r^ | This study |
| pET28a-*tagH*-BL21（DE3） | BL21 (DE3) complemented with pET28a-*tagH*, Kan^r^ | This study |
| pET28a-*hcp*-BL21（DE3） | BL21 (DE3) complemented with pET28a-*hcp*, Kan^r^ | This study |
| **Plasmids** |  |  |
| pET28a | Expression vector with N-6×His, N-Thrombin, N-T7, C-6×His tag, Kan^r^ | Lab collections |
| pET28a-*tagH* | pET28a expressing TagH tagged with 6×His | This study |
| pET28a-*hcp* | pET28a expressing Hcp tagged with 6×His | This study |
| pCold TF | Expression vector with N-His, N-Trigger factor, N-HRV 3C, N-Thrombin, N-Factor Xa tag, Amp^r^ | Lab collections |
| pCold TF-*hlyA* | pCold TF expressing HlyA | This study |
| pWM91 | Suicide plasmid; oriR oriT lacZ tetAR sacB, Amp^r^ | Lab collections |
| pWM91-*ΔtssB* | pWM91 carrying upstream and downstream fragments flanking *tssB*, Amp^r^ | This study |
| pWM91-*ΔtssM* | pWM91 carrying upstream and downstream fragments flanking *tssM*, Amp^r^ | This study |
| pWM91-*ΔtagH* | pWM91 carrying upstream and downstream fragments flanking *tagH*, Amp^r^ | This study |
| pWM91-*ΔprtV* | pWM91 carrying upstream and downstream fragments flanking *prtV*, Amp^r^ | This study |
| pWM91-*ΔhlyA* | pWM91 carrying upstream and downstream fragments flanking *hlyA*, Amp^r^ | This study |
| pWM91-*ΔhlyU* | pWM91 carrying upstream and downstream fragments flanking *hlyU*, Amp^r^ | This study |
| pBAD24 | Expression vector with araBAD promoter and rrnB T1 terminator, Amp^r^ | Lab collections |
| pBAD24-*tagH* | pBAD24 expressing TagH , Amp^r^ | This study |
| pBAD24-*tagH*^S38^*^A^* | pBAD24 expressing TagH^S38A^, Amp^r^ | This study |
| pBAD24-*tagH*^K54A^ | pBAD24 expressing TagH^K54A^, Amp^r^ | This study |
| pBAD24-*tagH*^S38AK54A^ | pBAD24 expressing TagH^S38AS38AK54A^, Amp^r^ | This study |
| pBAD24-*prtV* | pBAD24 expressing PrtV, Amp^r^ | This study |
| pBAD24-*fur* | pBAD24 expressing Fur, Amp^r^ | This study |
| pHRP309 | Promoter-cloning vector, Gm^r^ | Yiquan Zhang, Jiansu University |
| pHRP309-*nluc* | pHRP309 with *nluc*, Gm^r^ | This study |
| pHRP309*-nluc*-*hlyA* | pHRP309 *-nluc* with *hlyA* promoter region, Gm^r^ | This study |
| pHRP309-*nluc*-*hlyU* | pHRP309 *-nluc* with *hlyU* promoter region, Gm^r^ | This study |
| pHRP309-*nluc*-*fur* | pHRP309 *-nluc* with *fur* promoter region, Gm^r^ | This study |

**Supplementary Table 3 Primers used in this study**

| **Name** | **Primer sequence (forward/reverse, 5’ to 3’)** | **Use and description** |
| --- | --- | --- |
| **Primers for mutant construction** | | |
| *tagH* up-F | CAGCATACGTGGACCTCGTCAG | For construction of deletion mutant of *∆tagH* |
| *tagH* up-R | CTTCTGACGTGGCGAACTGCATATCATCCAGTAAGGAGGTCGAACTG |  |
| *tagH* down-F | CAGTTCGACCTCCTTACTGGATGATATGCAGTTCGCCACGTCAGAAG |  |
| *tagH* down-R | TCGTAGTCGGACGACATAAACATCG |  |
| *tssB* up-F | CCCTCGAGCCAAAATAGCCAAAAGCAACTG | For construction of deletion mutant of *∆tssB* |
| *tssB* up-R | CTTACGCTTGTGGCTCTTCTTGCGTCGCCGGAATATACTTGAT |  |
| *tssB* down-F | ATCAAGTATATTCCGGCGACGCAAGAAGAGCCACAAGCGTAAG |  |
| *tssB* down-R | CGGGATCCCGATACCAAAGAATTCAGGACCT |  |
| *tssM* up-F | CCCTCGAGTGGTTTGAAGGACAGTGGATGA | For construction of deletion mutant of *∆tssM* |
| *tssM* up-R | GTTCGGTAAATGGGTTGGCAATGGCAACGTTCAGCAAAATG |  |
| *tssM* down-F | CATTTTGCTGAACGTTGCCATTGCCAACCCATTTACCGAAC |  |
| *tssM* down-R | CGGGATCCAAACCCGACCCCTTCAAAGT |  |
| *hlyU* up-F | CCGCTCGAGTTGGAACAGTTAGCACAGC | For construction of deletion mutant of *∆tagH∆hlyU* |
| *hlyU* up-R | ATAAAGACGGTGCAACAGTTAACATACATAAAATTTGCAGTC |  |
| *hlyU* down-F | GACTGCAAATTTTATGTATGTTAACTGTTGCACCGTCTTTAT |  |
| *hlyU* down-R | CGCGGATCCAAAAAGAATACGTCAGCACTG |  |
| *prtV* up-F | ATAGCGGCCGCTACCAAAACCGCTCACACCA | For construction of deletion mutant of *∆tagH∆prtV* |
| *prtV* up-R | CCATTCGGCAGTGTCCATTCTCATTCACCACGCCTAAAT |  |
| *prtV* down-F | ATTTAGGCGTGGTGAATGAGAATGGACACTGCCGAATGG |  |
| *prtV* down-R | TATCTCGAGTCTCAGGCTTGGGTGAAACG |  |
| *hlyA* up-F | CGCGGATCCTTCAGTAAGTATGTGGTGGC | For construction of deletion mutant of *∆tagH∆hlyA* |
| *hlyA* up-R | AACCAATCGTCAGACCAAACTAAGCTCGGTAATGCGTTG |  |
| *hlyA* down-F | CAACGCATTACCGAGCTTAGTTTGGTCTGACGATTGGTT |  |
| *hlyA* down-R | CCGCTCGAGCTGGGATATCTTCGGTTCT |  |
| **Primers for constructs for complementation** | | |
| *tagH* -F | GGAATTCATGAACTCAGTGACATTACCTTCGT | For cloning complete length of *tagH* into pBAD24 |
| *tagH* -R | GAAGCTTTTATAGCTCCAGTTGCTTCTCGC |  |
| *prtV* -F | GGAATTCATGAAAACGATCAAAAAAACGCTATTAGC | For cloning complete length of *prtV* into pBAD24 |
| *prtV* -R | GAAGCTTTTACAGTTTGACTTTGATGGTGATGGTG |  |
| *fur* F | CCGGAATTCATGTCAGACAATAACCAAGCGCT | For cloning complete length of *fur* into pBAD24 |
| *fur* R | CCCAAGCTTTTATTTCTTCGGCTTGTGAGCGT |  |
| **Primers for constructs for expressing protein** | | |
| 28a-*tagH-*F | CCGCTCGAGATGAACTCAGTGACATTACCTTCGT | For cloning *tagH* into pET28a plasmid |
| 28a-*tagH-R* | CGCGGATCCTTATAGCTCCAGTTGCTTCTCGC |  |
| 28a-*hcp-*F | CCCTCGAGATGCCAACTCCATGTTATATCTCTATCGAA | For cloning *hcp* into pET28a plasmid |
| 28a-*hcp-*R | CGGGATCCTTACGCTTCGATTGGCTTACGC |  |
| TF-*hlyA-*F | TTCCATATGTTAGTTCAAATCAAATTGAACCCCTTT | For cloning *hlyA* into pCold TF plasmid |
| TF-*hlyA*-R | CGGAATTCATGCCAAAACTCAATCGTTGC |  |
| **Primers for constructs used in luminescence assay** | | |
| pHRP309-*nluc*-F | CGGAATTCATGGTCTTCACACTC | For cloning *nluc* into pHRP309 plasmid |
| pHRP309-*nluc*-R | CCAAGCTTTTACGCCAGAATGCGTTC |  |
| pHRP309-*nluc-hlyA*-F | GCGTCGACCAATCTATGCTTATACGG | For cloning *hlyA* promoter region into pHRP309-*nluc* plasmid |
| pHRP309-*nluc-hlyA*-R | CGGAATTCGCAACGATTGAGTTTTGG |  |
| pHRP309-*nluc-hlyU*-F | GCGTCGACTGTTAGTTCCAGGCAGTC | For cloning *hlyU* promoter region into pHRP309-*nluc* plasmid |
| pHRP309-*nluc-hlyU*-R | CGGAATTCTTTTAATTCCAACCCATTC |  |
| pHRP309-*nluc-fur*-F | GCGTCGACGCATCAAGGCATAAACGG | For cloning *fur* promoter region into pHRP309-*nluc* plasmid |
| pHRP309-*nluc-fur*-R | CGGAATTCATACTTTCCTGTTGATGTTC |  |
| Underline: Restriction endonuclease site | | |
| **Primers for construction for point mutation** | | |
| TagH-S38A-F | CAGGTGGGGTGATCGGC**GCA**TCGCCGAATGCGCAGTGGCGT | For construction of TagH mutant S38A in pBAD24 |
| TagH-S38A-R | ACGCCACTGCGCATTCGGCGA**TGC**GCCGATCACCCCACCTG |  |
| TagH-K54A-F | GATGCGCAAGGCAGTGTA**GCA**CCCATGCACTGTGAAGTGAT | For construction of TagH mutant K54A in pBAD24 |
| TagH-K54A-R | ATCACTTCACAGTGCATGGG**TGC**TACACTGCCTTGCGCATC |  |
| TagH-S38AK54A-F | GATGCGCAAGGCAGTGTA**GCA**CCCATGCACTGTGAAGTGAT | For construction of TagH mutant S38AK54A in pBAD24-*tagH*^S38A^ |
| TagH-S38AK54A-R | ATCACTTCACAGTGCATGGG**TGC**TACACTGCCTTGCGCATC |  |
| Bold: Alanine substituted site | | |
| **Primers for Real time PCR** | | |
| q-*hlyA*-F | CAAGCAGAGATGCAAGCCCA | For qRT-PCR to targeting *hlyA* |
| q-*hlyA*-R | TCGCACGGTTGACACTGATG |  |
| q-*hlyU*-F | CTCAGCCAATCTGCTCTT | For qRT-PCR to targeting *hlyU* |
| q-*hlyU*-R | AGTTCAATCATCGCCTTC |  |
| q-*fur*-F | AGCCAGAGTGCCAACATATTAG | For qRT-PCR to targeting *fur* |
| q-*fur*-R | AATACTGACTTGCCGCCTTC |  |
| q-*prtV*-F | TACTTGACGGATGGCGGTCT | For qRT-PCR to targeting *prtV* |
| q-*prtV*-R | CGTCCACATACCACACCAGC |  |
| q-16s-F | CGGTAATACGGAGGGTGCAA | For qRT-PCR to targeting 16srDNA |
| q-16s-R | CACCTGCATGCGCTTTACG |  |
